# MACC1-Dependent Antitumor Effect of Curcumin in Colorectal Cancer

**DOI:** 10.3390/nu14224792

**Published:** 2022-11-12

**Authors:** Nazli Güllü, Janice Smith, Pia Herrmann, Ulrike Stein

**Affiliations:** 1 Department Translational Oncology of Solid Tumors, Experimental and Clinical Research Center, Charité—Universitätsmedizin Berlin, and Max-Delbrück-Center for Molecular Medicine, Robert-Rössle-Straße 10, 13125 Berlin, Germany; 2 German Cancer Consortium (DKTK), Heidelberg, Im Neuenheimer Feld 280, 69120 Heidelberg, Germany

**Keywords:** curcumin, MACC1, colorectal cancer, cancer stemness

## Abstract

Metastasis is the main reason for the high mortality rate of colorectal cancer (CRC) patients. Despite the whole improvement in the field of cancer medicine, the treatment options for the patient in the late stages are very restricted. Our previous studies have elucidated metastasis-associated in colon cancer 1 (MACC1) as a direct link to metastasis formation. Therefore, we have aimed to inhibit its expression by using natural products, which are recently the center of most studies due to their low side effects and good tolerability. In this study, we have investigated the effect of one of the promising natural products, curcumin, on MACC1 expression and MACC1-induced tumor-promoting pathways. Curcumin reduced the MACC1 expression, restricted the MACC1-induced proliferation, and was able to reduce the MACC1-induced cell motility as one of the crucial steps for the distant dissemination of the tumor. We further showed the MACC1-dependent effect of curcumin on clonogenicity and wound healing. This study is, to our knowledge, the first identification of the effect of curcumin on the restriction of cancer motility, proliferation, and colony-forming ability by using MACC1 as a target.

## 1. Introduction

Cancer is one of the leading causes of death worldwide. Only in 2020, 19 million patients were diagnosed with cancer. Among them, 40% developed metastasis, and 10 million lost their lives [1]. One of the most common cancer types is colorectal cancer (CRC) [2]. Although recent studies report improvements in the early stages, the overall survival rate of patients in the late stages is less than 10% [3]. Accordingly, it is crucial to identify novel biomarkers that can be used for metastasis prediction and simultaneously as a target of therapeutic interventions to treat patients at high risk for metastasis. One of the remarkable biomarkers, metastasis-associated in colon cancer 1 (MACC1), was discovered in 2009 [4]. The high expression of this gene drops the metastasis-free survival rate and, correlatively, the overall survival rate of the patients drastically [4]. Numerous research groups have acquainted its importance as a metastasis inducer, prognostic, and predictive biomarker for more than 20 different tumor entities, including CRC, breast cancer, and hepatocellular carcinoma [5,6,7,8,9]. MACC1 promotes cell death, genome instability, sustaining proliferative signaling, and inflammation. Furthermore, the high expression of MACC1 induces stemness, migration, invasion, and metastasis [6,10,11,12,13,14,15,16]. These studies center MACC1 as a robust therapeutic target to restrict cancer progression and distant tumor dissemination.

Previous studies tried to develop treatment options to restrict metastasis formation; however, the inadequate outcomes forced the establishment of new treatment modalities. Therefore, recent studies endeavor to improve the standard therapy by establishing natural compound-based treatment or combining natural products with approved drugs [17]. In previous years, the effect of saffron on the remission of metastasis and the inhibition of MACC1-promoted migration and proliferation was published [18,19]. However, saffron treatment is not able to reduce the MACC1 expression. Correlatively the MACC1-dependent effect of saffron is restricted and cannot entirely inhibit the tumor-promoting and metastasis-leading effect of MACC1. Therefore, among other natural products, we investigated the MACC1-dependent effect of curcumin, which is accounted as one of the most promising natural compounds to restrict cancer progression [20].

Curcumin is the curcuminoid compound of turmeric (Curcuma longa). Its remedial effect was shown for various diseases, such as Alzheimer’s and gastrointestinal disorders [21,22,23]. Recent studies also demonstrated curcumin’s inhibitory impact on cancer progression and distant dissemination of tumors [24]. Curcumin is able to inhibit proliferation and induces apoptosis by increasing the expression of wild-type p53 [25]. Further studies revealed that curcumin treatment enhances G1/S cell cycle arrest by the downregulation of cyclin D1 [26]. Moreover, curcumin treatment induces autophagy through the suppression of the AKT/mTOR/p70S6K pathway and the activation of ERK1/2 [27]. In addition to these, its effect on angiogenesis, cancer stemness, and NFκB signaling was reported in different tumor entities [13,28]. Preliminary studies indicated the potential inhibitory effect of curcumin on prostate cancer metastasis by decreasing the expression of CXCL1 and 2 [29]. 

Despite all the studies, curcumin’s entire anti-cancer effects are still unclear. Therefore, in this study, we investigated the effect of curcumin on MACC1-driven tumor progression. To our knowledge, this is the first study showing a relationship between curcumin and MACC1.

## 2. Materials and Methods

### 2.1. Cell Lines and Compounds

The CRC cell lines SW620 and HCT116 were used for the experiments. The cells were obtained from the American Type Culture Collection (Manassas, VA, USA) maintained in DMEM and RPMI 1640 (both Thermo Fisher Scientific, Waltham, MA, USA), supplemented with 10% fetal bovine serum (FBS). The cells were preserved at 37 °C with 5% CO_2_ in a humidified incubator. 

For the experiments, previously by Crispr-Cas9-technology-generated MACC1 knocked-out clones of SW620 and HCT116 were used: SW620/KO-MACC1, HCT116/KO-MACC1 (control: SW620/KO-Control, HCT116/KO-Control).

In addition, various curcumin concentrations (50 μM–1.5 μM) were prepared freshly by diluting the curcumin powder in DMSO. At the same time, as a negative control, the cells were treated with DMSO and named untreated.

### 2.2. RNA Extraction and Quantitative PCR

The Gene Matrix Universal RNA purification Kit was used to isolate total RNA according to the manufacturer’s instructions (Roboklon, Gdansk, Poland). The extracted RNAs were used either immediately after the extraction or maintained at −80 °C. The concentration and the quality of RNA were assessed with NanoDrop 2000 (Thermo Fisher Scientific, Waltham, MA, USA). The total RNA (50 ng) was reverse transcribed by using the Biozym kit (Biozym, Hessisch Oldendorf, Germany).

For the amplification of the cDNAs, the Syber green format (Biozym, Hessisch Oldendorf, Germany) was used. The amplification protocol was performed for 10 min at 95 °C and 45 cycles of 10 s at 95 °C, 30 s at 60 °C, and 4 s at 72 °C. The gene expressions were normalized to GAPDH. The mean values of the duplicates were calculated using standard curves. The primer sequences were listed in Supplementary Table S1.

### 2.3. Protein Extraction and Western Blot

1 × 10^6^ cells were seeded in 10 cm Petri dishes. After 24 h, the cells were treated with the respective curcumin concentrations, and after 24 h, the cells were lysed with RIPA buffer (50 mM Tris–HCl; pH 7.5, 150 mM NaCl, and 1% Nonidet P-40, supplemented with complete protease inhibitor tablets; Roche Diagnostics, Mannheim, Germany). According to the manufacturer’s instructions, the protein concentration was measured using the Pierce BCA Protein Assay (Thermo Fisher Scientific, Waltham, MA, USA). To detect the protein levels, 30 μg of protein was resolved by electrophoresis on 10% sodium dodecyl sulfate-polyacrylamide gels. The proteins were transferred to nitrocellulose membranes, blocked with tris-buffered saline-tween20 (TBS-T) containing 5% skim milk powder and incubated with MACC1 primary antibody (Thermo Fisher Scientific, Waltham, MA, USA dilution 1:1000) overnight at 4 °C. On the next day, horseradish peroxidase (HRP)-labeled anti-rabbit IgG (Promega, Fitchburg, WI, USA dilution 1:10,000) or anti-mouse IgG (Thermo Fisher Scientific, Waltham, MA, USA dilution 1:10,000) secondary antibodies were added. The proteins were visualized by using the chemiluminescent reagent WesternBright (Advansta, San Jose, CA, USA); and subsequently exposed to CL-Xposure Films (Thermo Fisher Scientific, Waltham, MA, USA); as a loading control, ß-actin (Thermo Fisher Scientific, Waltham, MA, USA) was used.

### 2.4. Live Cell Imaging

7.5 × 10^3^ cells were seeded in 96-well plates to investigate the curcumin’s effect on cell proliferation. Cells were treated with different curcumin concentrations between 7.5 μM–30 μM. The plates were placed into the IncuCyte system (Essen Bioscience, Ann Arbor, MI, USA). Cell proliferation was assessed every 2 h for 72 h and analyzed using the integrated software (Essen Bioscience, Ann Arbor, MI, USA).

### 2.5. Viability Assay

Cell viability was detected by using an MTT assay. First, 5 × 10^4^ control or curcumin-treated cells were seeded into the 96-well plates. After 24 h or 48 h, the cells were incubated with MTT solution for 2.5 h at 37 °C. Afterward, the medium was removed, and the crystals were dissolved in DMSO. The absorbance was measured at 560 nm using a multi-well plate reader. 

### 2.6. Migration Assay

The migration capability of the cells was determined using the Boyden chamber. The serum-starved cells (6 h 0.5% FBS) were seeded into a 12 mm diameter transwell upper chamber, and 10% FBS-containing medium served as a chemoattractant in the lower chamber. Curcumin was added to both chambers. Subsequently, the cells that migrated towards the down part were collected after 16 h. The collected cells were counted using a Guava live-cell counter (Luminex, Saluggia, Italy). 

### 2.7. Wound Healing Assay

30 × 10^3^ cells were seeded into a 96-well plate. After 12 h, the wounds were created with the wound-healing insert (Essen Bioscience, Ann Arbor, MI, USA). The medium was replaced with solvent and various curcumin concentrations. Subsequently, plates were placed into the IncuCyte live-cell-imaging system (Essen Bioscience, Ann Arbor, MI, USA). The wound was visualized every 2 h with the scratch mask of the integrated program. After 48 h, the wound size was compared to the wound size starting day 0.

### 2.8. Clonogenic Assay

A clonogenicity assay was performed to assess the ability of the single cells to grow into colonies. The 400 cells/well were seeded into 6-well plates and allowed to attach for 24 h. After adding (drug-containing-) fresh medium, they were incubated for 10 days in a humidified incubator at 37 °C with 5% CO_2_. For the analysis and visualization of the colonies, cells were fixed and stained with 1% Formaldehyde and 0.1% crystal violet containing PBS. The colonies were visualized by the FlourChemQ system (Alpha Innotech, Cell Biosciences, Santa Clara, CA, USA), and numbers and areas were determined using a colony plug of integrated AlphaView software version 2.0.1.1. (Alpha Innotech, Cell Biosciences, Santa Clara, CA, USA).

### 2.9. Statistics

All of the statistical analyses were performed with GraphPad Prism version 9.0 (La Jolla, CA, USA). The comparison of multiple groups was evaluated by one-way analysis of variance ANOVA. *p* values smaller than 0.05 were considered statistically significant (* = *p* < 0.05, ** = *p* < 0.01, *** = *p* < 0.001, **** = *p* < 0.0001).

## 3. Result

### 3.1. Curcumin Treatment Reduced the MACC1 Expression

**Figure 1:**
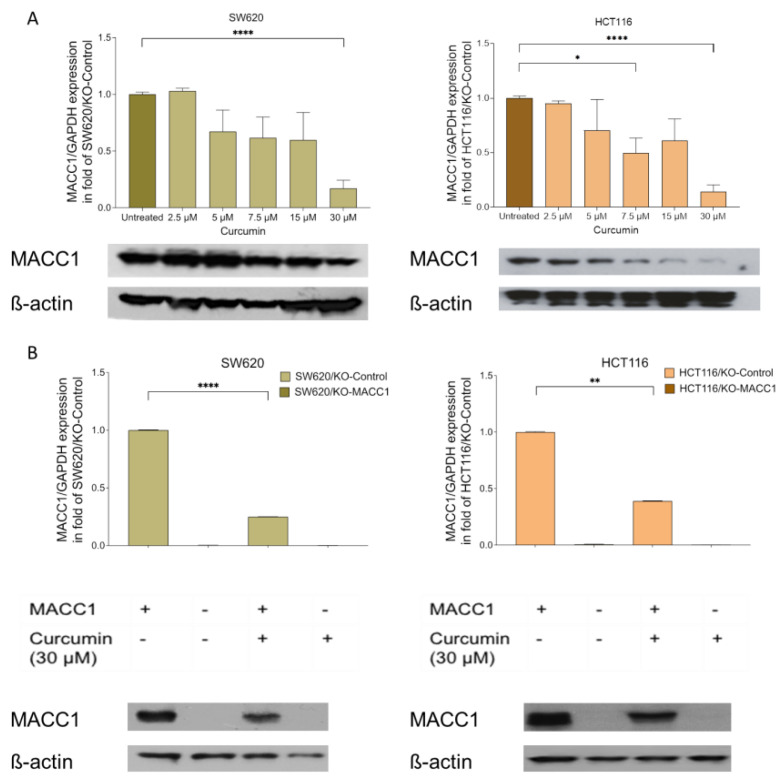
Curcumin treatment reduced the metastasis-associated in colon cancer 1 (MACC1) expression. Colorectal cancer (CRC) cells SW620 and HCT116 cells were treated with various concentrations of curcumin. MACC1 mRNA expression was determined by qRT-PCR, and protein levels were detected with Western blot analysis. MACC1 mRNA and protein levels decreased upon increasing curcumin concentration in both SW620 and HCT116 cells (**A**). The same experiment was performed using the MACC1 knock-out clones, whereas the curcumin treatment did not impact the knock-out clones (**B**). MACC1 mRNAs were normalized with GAPDH. ß-actin has been used as a loading control for the Western blot analysis. Data represent mean ± SEM (*n* ≥ 3), * = *p* < 0.05, ** = *p* < 0.01, **** = *p* < 0.0001.

Initially, we determined the effect of curcumin on MACC1 expression. The SW620 cells were treated with various concentrations of curcumin. The endogenous MACC1 expression in the SW620 cells was reduced by 33% under 5 μM curcumin treatment compared to the untreated cells. The 7.5 μM, 15 μM, and 30 μM curcumin treatments decreased the MACC1 expression by 39%, 58%, and 83%, respectively (Figure 1A). The MACC1 protein levels dropped under 7.5 μM and higher curcumin treatments compared to untreated cells (Figure 1A). 

These results were further consolidated in the HCT116 cells. The 7.5 μM curcumin treatment reduced the MACC1 expression by more than 39% (Figure 1A). The highest effect was observed at 30 μM curcumin treatment, whereby the MACC1 expression was reduced by more than 75%. The treatment of 7.5 μM and higher curcumin concentrations for 24 h reduced the MACC1 protein level in both cell lines (Figure 1B). Although various curcumin concentrations were able to reduce the MACC1 expression, the decrease in the MACC1 mRNA and protein levels is more prominent in treatments with higher curcumin dosages.

### 3.2. Curcumin Reduced the MACC1-Induced Proliferation Rate and Viability 

**Figure 2:**
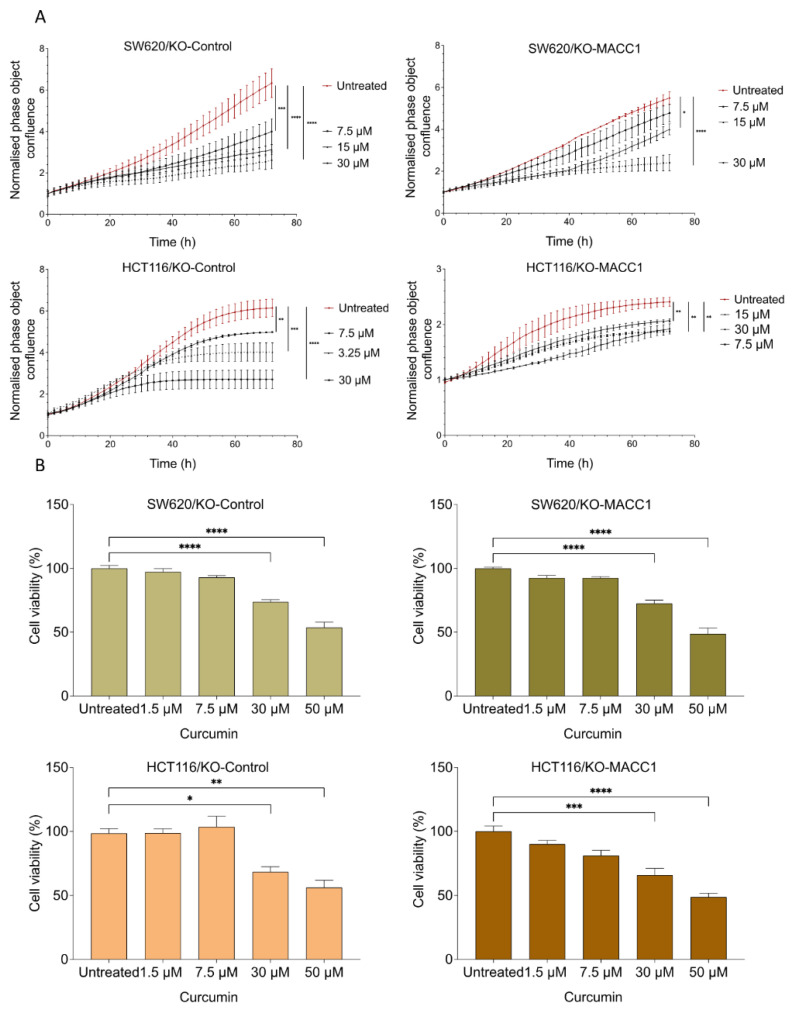
Curcumin reduced proliferation and viability of CRC cell lines with high or moderate MACC1 expression. CRC cells were treated with various concentrations of curcumin. Every 2 h, the confluency of the cells was assessed by a cell imaging system. Curcumin significantly reduced the proliferation of the cells with high or moderate MACC1 expression (SW620/KO-Control, HCT116/KO-Control) (**A**). Cell viability was determined using the MTT assay. Viability of the CRC cells was reduced upon curcumin treatment in a dose-dependent manner (**B**). Data represent mean ± SEM (*n* ≥ 3), * = *p* < 0.05, ** = *p* < 0.01, *** = *p* < 0.001, **** = *p* < 0.0001.

CRC cell lines SW620, HCT116, and their MACC1 knock-out clones were treated with various curcumin concentrations, and cell confluency was measured label-free for 72 h. Every 2 h, the confluency of the cells was monitored by using the IncuCyte live cell imaging system. SW620 cells with high endogenous MACC1 expression showed by 33.56% higher proliferation rate compared to its MACC1 knock-out clones. Once endogenously high MACC1-expressing SW620 cells were treated with 7.5 μM, 15 μM, and 30 μM curcumin for 72 h, they showed a reduced proliferation rate by 34%, 54%, and 62%, respectively, compared to the untreated SW620/KO-Control cells (Figure 2A). On the contrary, 7.5 μM, 15 μM, and 30 μM curcumin treatment for 72 h reduced the proliferation of SW620/KO-MACC1 cells only by 13%, 24%, and 58% compared to the untreated SW620/KO-MACC1 cells.

The same effect was also observed in the HCT116 cells. The HCT116/KO-Control cells proliferated 2.5 times more compared to the MACC1 knock-out cells. The treatment with 30 μM curcumin reduced the proliferation rate by 56%. Further treatment with 7.5 μM and 15 μM reduced the proliferation rate by 19% and 34.84%, respectively, compared to the untreated cells (Figure 2A). Especially, the proliferation of the cells with high MACC1 expression was reduced even under treatment of lower curcumin concentrations in both cell lines. 

Moreover, the effect of curcumin on viability was investigated. The treatment with 50 μM and lower curcumin concentrations for 24 h and 48 h significantly reduced the viability of the cells (Figure 2B and Figure S1). The viability of the SW620/KO-Control and the HCT116/KO-Control cells dropped more than 40%.

### 3.3. Curcumin Treatment Restricted the Migratory Capability of the CRC Cells

**Figure 3:**
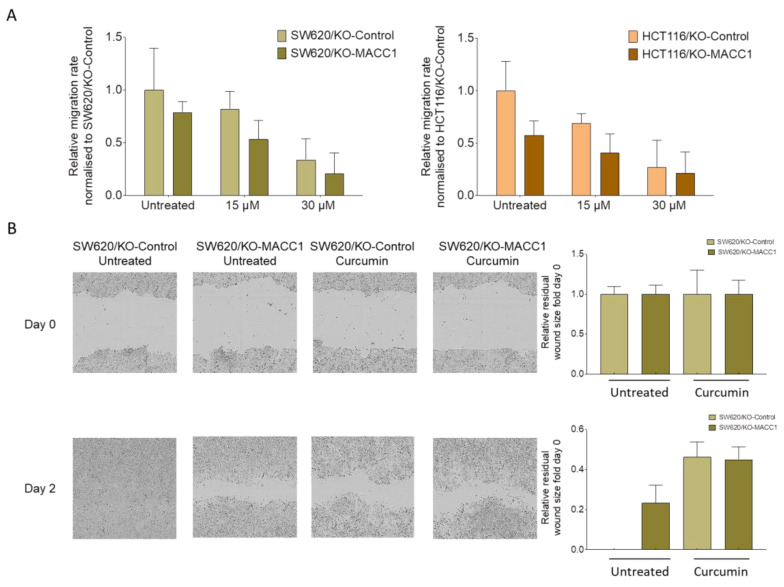
Curcumin reduced the cell motility in CRC cell lines with high or moderate MACC1 expression. SW620 and HCT116 cells were treated with 15 μM and 30 μM curcumin, and their impact on the migratory capability was measured by Boyden chamber assay (**A**). The results were further validated by a wound healing assay, and wound size was analyzed and visualized by the IncuCyte live cell imaging system (**B**). Data represent mean ± SEM (*n* ≥ 3).

In the context of the distant dissemination of the tumor, we investigated the effect of curcumin on the migratory capability of CRC cells. For this, we used the Boyden chamber assay. The high endogenous expression of MACC1 increased the migration rate of the SW620 cells by 22% compared to its MACC1 knock-out clones (Figure 3A). SW620/KO-Control cells migrated 66% less under 30 μM curcumin treatment. Similar effects were determined in MACC1 moderately-expressing HCT116 cells. Curcumin treatment reduced the migration rate of the HCT116/KO-Control cells by more than 73% under 30 μM curcumin treatment compared to untreated cells (Figure 3A).

We further complemented the effect of curcumin on directed migration in a wound-healing assay. Once the SW620/KO-Control cells were exposed to curcumin, the wound closed slower compared to the untreated cells, as shown by the representative figures and the corresponding quantification (Figure 3B).

### 3.4. Curcumin Restricted the Colony-Forming Capability of the Cells 

**Figure 4:**
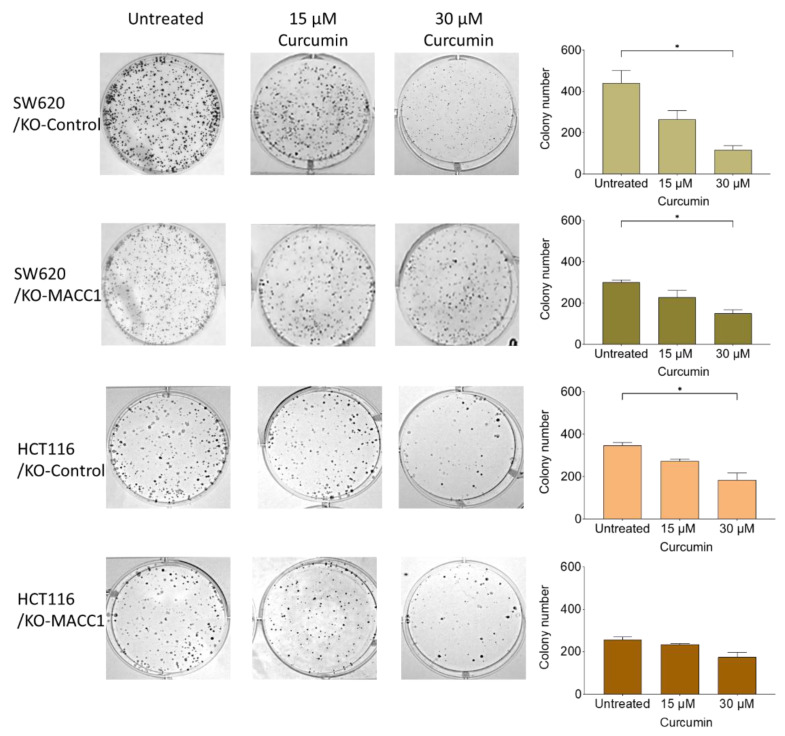
Curcumin reduces MACC1-mediated clonogenicity. 400 cells/well were seeded into 6-well plates and were treated with various curcumin concentrations. After 10 days, the cells were fixed and stained with crystal violet. Afterward, the cells were imaged, and the colony numbers were assessed. Data represent mean ± SEM (*n* ≥ 3), * = *p* < 0.05.

A clonogenic assay was performed to assess the effect of curcumin on reproductive viability, the ability to divide limitlessly, and the capability of the cells to form de novo colonies. SW620 cells with MACC1 expression showed an increased colony number compared to knock-out clones. SW620 cells formed 1.3 times more colonies compared to MACC1 knock-out clones, whereas moderate MACC1 expressing HCT116 cells formed 1.3 times more colonies compared to HCT116/KO-MACC1 cells (Figure 4). Once SW620/KO-Control cells were exposed to 30 μM curcumin, their clonogenicity dropped by more than 70%. The same effect was elucidated by the HCT116/KO-Control cells. The treatment of 15 μM and 30 μM curcumin reduced the clonogenicity rate by 21% and 42%, respectively. The decrease in the colony-forming rate of the HCT116/KO-MACC1 cells was restricted by 31% under 30 μM curcumin treatment (Figure 4). 

## 4. Discussion

Among all cancer types, CRC accounted for the second most common and third deadliest cancer [1]. Therefore, it is crucial to establish new treatment opportunities by establishing novel biomarkers that can be used for the stratification of the course of the disease and as a target for the restriction of cancer- and metastasis formation. In this study, we investigated the effect of the natural compound curcumin on reversing the tumor-promoting effect of MACC1 by reducing its expression. 

In previous studies, the effect of statins on reducing MACC1 expression was demonstrated [30,31]. Statins (HMG-CoA reductases) are mainly used to reduce cholesterol levels and correlatively treat cardiovascular diseases [32,33]. The current studies broadened the impact of statins and showed their effect on other diseases, such as cancer and Alzheimer`s disease [34,35,36,37]. The effect of statins on the remission of cancer and on the improvement of the survival rate of CRC patients were revealed in a transatlantic cohort [31]. However, their effects are broad, and they were involved in various essential pathways. Therefore, it led to severe side effects, and their usage is still under consideration for ALS, HIV, some cardiomyopathic patients, and especially for patients older than 80 years [38,39,40,41]. 

We further elucidated the inhibitory effect of saffron on MACC1-dependent proliferation and migration; despite its MACC1-dependent effect, saffron treatment did not reduce MACC1 expression [18]. Especially due to the high costs and low availability of saffron, we studied further the effect of curcumin on MACC1. Recent studies showed the impact of curcumin on different metastasis-promoting pathways, such as NFκB and MMPs, in various tumors [13,42,43]. Most importantly, a wide range of studies showed that curcumin could be tolerated even in high dosages [44]. 

The curcumin treatment reduced the MACC1 expression in a concentration-dependent manner in the endogenously high/mild MACC1-expressing SW620/HCT116 cells. The reduction was more prominent under the treatment of the higher dosages (15 μM and 30 μM). To elucidate the MACC1-dependent effect of curcumin, we further treated the MACC1 knock-out clones of the same cells.

Previous studies reported that high MACC1 expression increases the proliferation and viability of a wide range of tumor types, including CRC and breast cancer [14]. Therefore, we investigated if curcumin reduces MACC1 expression and further reverses MACC1-promoted proliferation and viability. Exposure to various curcumin concentrations reduced the proliferation rate of the CRC cells. However, this reduction was more prominent by the endogenously high/moderate MACC1-expressing cells. Even exposure to lower concentrations (7.5 μM) for 72 h could significantly reduce the proliferation of MACC1-expressing cells. The same effect was not observed by the SW620/KO-MACC1 clones. These results were further validated in viability assay.

The distant dissemination of the tumor requires the motility capability of the cells and the ability to form de novo tumors at a distant site [45,46]. It is known that MACC1 expression increases this process by inducing several pathways, including PI3K/AKT and ß-catenin [6,16,47,48]. Therefore, we have exposed the cells to various curcumin concentrations, showing that the migration reduced upon treatment. We were aware that curcumin has a general anti-migratory effect [49]; however, in this study, we demonstrated that this reduction was more prominent in the MACC1-expressing cells. We further observed a similar effect of curcumin on directed migration by a wound-healing assay. MACC1 expression increased the wound-healing rate, but once the cells were treated with curcumin, the MACC1 accelerated wound-healing rate decreased. 

We further showed the MACC1-dependent effect of curcumin on clonogenicity. The de novo tumor-forming capability of the cancer cells is essential for metastasis formation. The high level of MACC1 enhances the clonogenicity of the tumor cells. Therefore, to inhibit the MACC1-induced clonogenicity, the cells were treated with various curcumin concentrations for 10 days. Although curcumin generally reduces clonogenicity [50], it showed an accelerated effect on the moderate/high MACC1-expressing cells compared to MACC1 knock-out clones.

This study successfully demonstrated curcumin’s novel effect on MACC1 expression and the inhibition of MACC1-induced pro-tumorigenic pathways, including proliferation, migration, wound healing, and clonogenicity in established cell lines. However, we do not know how curcumin elicits its effect on MACC1 expression. Previous studies showed that curcumin treatment inhibits ERK phosphorylation, which promotes MACC1 expression [6,51]. This interaction should be further investigated.

Taken together, we illustrated the prominent inhibitory effect of curcumin on MACC1 expression and MACC1-induced phenotypes. In addition, we further revealed the MACC1-dependent anti-proliferative and anti-migratory effects of curcumin. Although the experiments have been conducted in the established cell lines, these promising results can expand in the other models so that curcumin can further supplement the treatment of CRC patients, notably those who cannot tolerate statins and have high MACC1 expression.

## 5. Conclusions

This is the first study showing the effect of curcumin on reducing MACC1 expression in the established cell lines. In this study, we demonstrated the MACC1-dependent inhibitory effect of a wide range of curcumin concentrations on MACC1-induced viability, proliferation, migration, wound healing, and clonogenicity. This study provides evidence to target MACC1 via natural products to establish new treatment opportunities for the treatment of especially MACC1-driven tumor progression and metastasis. 

## Data Availability

All relevant data is contained within the manuscript.

## Supplementary Materials

The following supporting information can be downloaded at: https://www.mdpi.com/article/10.3390/nu14224792/s1, Table S1: Primers used for RT-qPCR; Figure S1: Curcumin reduced viability of CRC cell lines with high or moderate MACC1 expression after 48 h.
