# Supplementary material for: MACC1-Dependent Antitumor Effect of Curcumin in Colorectal Cancer
Source: Nutrients. 2022 Nov 12;14(22):4792. doi: 10.3390/nu14224792 (PMC9692505; doi:10.3390/nu14224792)
Supplement: Supplementary file 1 [file nutrients-14-04792-s001.zip › nutrients-1993597-supplementary.pdf]

Supplementary Table S1: Primers used for RT-qPCR

Gene	Primer	Sequence
MACC1	Fwd	5`-TTC TTT TGA TTC CTC CGG TGA-3`
	Rev	5`-ACT CTG ATG GGC ATG TGC TG-3`
GAPDH	Fwd	5`-GTCTCCTCTGACTTCAACAGCG-3`
	Rev	5`-ACCACCCTGTTGCTGTAGCCAA-3`

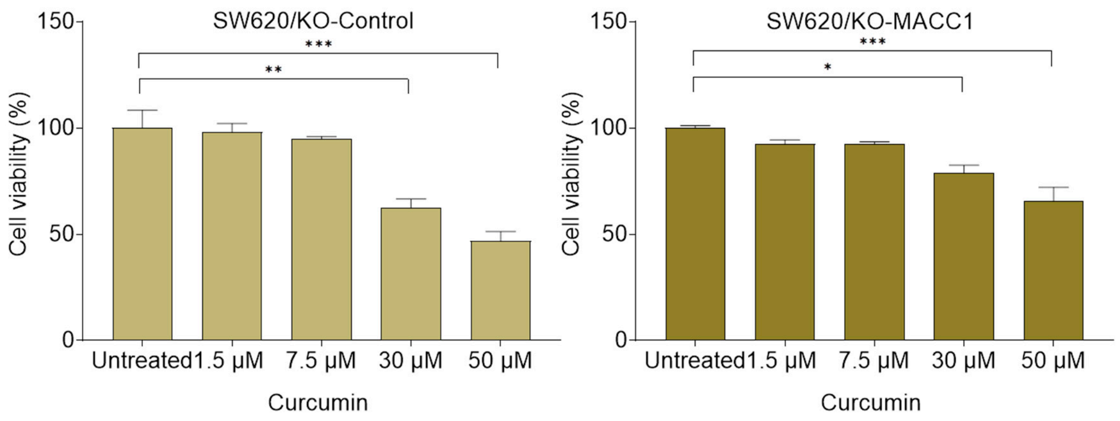

Supplementary Figure S1: Curcumin reduced viability of SW620 cells after 48 h. CRC cells were treated with various concentrations of curcumin. Cell viability has been further determined using the MTT assay. Viability of the CRC cells was reduced upon 48 h curcumin treatment in a dose-dependent manner. Data represent mean ± SEM ($n \geq 3$), *=$p < 0.05$, **=$p < 0.01$ ***=$p < 0.001$
